# Prevalence rates of mucopolysaccharidoses in Poland

**DOI:** 10.1007/s13353-014-0262-5

**Published:** 2014-12-04

**Authors:** Agnieszka Jurecka, Agnieszka Ługowska, Adam Golda, Barbara Czartoryska, Anna Tylki-Szymańska

**Affiliations:** 1 Department of Genetics, University of Gdańsk, ul. Wita Stwosza 59, 80-308 Gdańsk, Poland; 2 Institute of Psychiatry and Neurology, Warsaw, Poland; 3 Department of Cardiology, Gliwice General Hospital, Gliwice, Poland; 4 Department of Pediatrics, Nutrition and Metabolic Diseases, The Children’s Memorial Health Institute, Warsaw, Poland

**Keywords:** Mucopolysaccharidoses, Prevalence

## Abstract

The aim of this study was to determine the prevalence rates of mucopolysaccharidoses in Poland and to compare them with other European countries. A retrospective epidemiological survey covering the period between 1970 and 2010 was implemented. Multiple ascertainment sources were used to identify affected patients. The overall prevalence of mucopolysaccharidoses in the Polish population was 1.81 per 100,000. Five different mucopolysaccharidoses were diagnosed in a total of 392 individuals. MPS III was the most frequent mucopolysaccharidosis, with a birth prevalence of 0.86 per 100,000 live births. A prevalence of approximately 0.22 cases per 100,000 births was obtained for MPS I. For MPS II, the prevalence was estimated as 0.45 cases per 100,000 births; for MPS IV A and B as 0.14 cases in 100,000 births; and that for MPS VI as 0.03 cases per 100,000 births. 1. The prevalence pattern of mucopolysaccharidosis in Poland is lower when compared to the prevalence reported for other European countries, such as the Netherlands, Czech Republic, or Germany, but similar to countries like Sweden and Denmark. 2. Different frequencies of the various forms of mucopolysaccharidosis were observed. 3. In the case of MPS VI, the incidence values for Poland were the lowest of all the studies previously published so far.

## Introduction

The mucopolysaccharidoses represent the largest group of lysosomal storage disorders (LSDs) and are characterized by progressive multiorgan involvement, leading to severe disability and premature death. Each type results from a deficiency of a specific lysosomal enzyme that participates in the stepwise degradation of glycosaminoglycans (GAGs) (Neufeld and Muenzer 2001). Seven types of mucopolysaccharidosis disorders, caused by deficiencies of ten different enzymes, are as follows: MPS I (Hurler MIM 607014, Hurler–Scheie MIM 607015, Scheie MIM 607016), MPS II (Hunter syndrome MIM 309900), MPS III A (MIM 252900), B (OMIM 252920), C (MIM 252930), or D (MIM 252940) (Sanfilippo syndrome), MPS IV A (MIM 253000) or B (MIM 253010) (Morquio syndrome), MPS VI (Maroteaux–Lamy syndrome, MIM 253200), MPS VII (Sly disease, MIM 253220), and MPS IX (MIM 601492) (Neufeld and Muenzer 2001). There is a wide variety of clinical symptoms between and within each type (Neufeld and Muenzer 2001). Most mucopolysaccharidoses are inherited in an autosomal recessive manner, with the exception of mucopolysaccharidosis type II, which shows X-linked recessive inheritance.

A retrospective epidemiological survey study of the mucopolysaccharidoses was implemented to estimate the prevalence and incidence rates of the different types for the period from 1970 until 2010 in Poland. There have been a number of reports on the prevalence of particular disorders in select populations. One of the first studies on the frequency of mucopolysaccharidoses in the province of British Columbia (Canada) was carried out by Lowry and Renwick (1971). These data were updated by Lowry et al. (1990) and later by Applegarth et al. (2000). Further studies on the prevalence of the various types of mucopolysaccharidoses in different populations have shown considerable variation (Meikle et al. 1999; Poupetová et al. 2010; Poorthuis et al. 1999; Nelson et al. 2003; Malm et al. 2008; Baehner et al. 2005). As the development of therapies for this group of disorders proceeds and the possibilities for neonatal screening are explored, it becomes important to obtain accurate values for the prevalence of these disorders (Meikle et al. 1999). Treatment options for a number of mucopolysaccharidoses have rapidly expanded and currently include enzyme replacement therapy (ERT), substrate reduction, and hematopoietic stem cell transplantation (Hollak and Wijburg 2014; Jakóbkiewicz-Banecka et al. 2007). Current available treatment authorized in the EU includes ERT for MPS I, II, IV, and VI (Hollak and Wijburg 2014). These data will be required in order to accurately assess the cost of these disorders to public health care systems and will be a key factor in the adoption of screening and treatment programs (Meikle et al. 1999).

The aim of this study was to calculate the birth prevalence of mucopolysaccharidoses in Poland and to compare the results with reported epidemiologic data from other European countries.

## Materials and methods

### Patient enrolment and data collection

Data from individuals who had been diagnosed with mucopolysaccharidosis at the Department of Genetics, Institute of Psychiatry and Neurology in Warsaw between 1970 and 2010 have been analyzed. The Children’s Memorial Health Institute is the only laboratory providing diagnostic testing for mucopolysaccharidosis in Poland.

In order to obtain as complete an ascertainment as possible, multiple sources were utilized:

1. Patient records from the Department of Metabolic Diseases, The Children’s Memorial Health Institute, Warsaw, Poland.
2. Laboratory records from the Department of Genetics, Institute of Psychiatry and Neurology, Warsaw, Poland.
3. Membership list of the Polish MPS Society (patient support group).

The patient list, presumed to be exhaustive, was then used for the data collection.

### Diagnosis of mucopolysaccharidosis

The initial diagnosis of mucopolysaccharidosis was based on the demonstration of accumulated substrates (GAGs) in body fluids using electrophoretic methods. The definite diagnosis was made by demonstrating the deficiency of the relevant enzyme and/or presence of pathogenic mutation.

### Data analysis for prevalence rates

The live births numbers in Poland were obtained from the GUS Central Statistical Office (XXXX). For the years 1970–1990, in Poland, the live births numbers were available every five years, while for the years 1990–2009, the live births numbers were available annually. The missing live births values were calculated using linear interpolation for the years 1983–1989 and using the least squares approach to estimate the values for the year 2010 for Poland. The calculations of incidence rates with specification of the confidence interval were conducted using Windows Microsoft Excel 2007 software.

Confidence intervals (95 %) for the birth prevalences were estimated from the Poisson distribution using Simple Interactive Statistical Analysis (SISA) software (Uitenbroek 1997).

Carrier frequency was calculated using the Hardy–Weinberg equation (Strachan and Read 2004).

## Results

### Prevalence rates in Poland

**Fig. 1:**
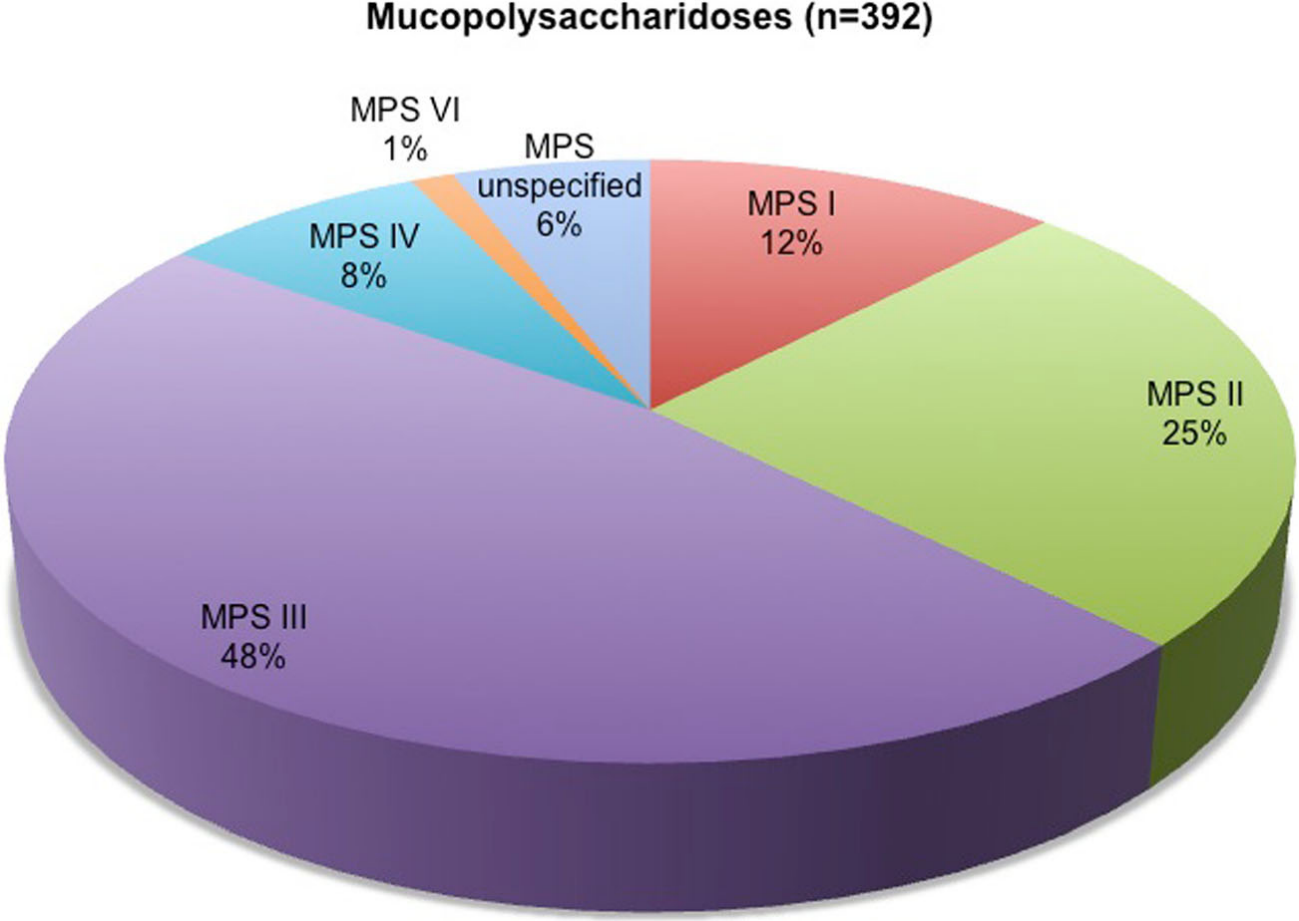
Relative rate of mucopolysaccharidoses in Poland

Live births registered at the Polish Bureau of Statistics in the period from 1970 until 2010 totalled 21,686,890. In the past four decades, a total of 392 patients have been diagnosed with one of seven specific types of mucopolysaccharidoses in Poland (Fig. 1). The number of diagnosed patients gradually increased during the study period, from less than 10 cases per year in the 1970s to 15–20 new cases diagnosed annually in recent years. The combined birth prevalence for all mucopolysaccharidoses is 1.81 per 100,000 live births. Data on the prevalence rates are presented in Table 1.

**Table 1:** Mucopolysaccharidoses: comparison of data in different populations

Disease	Poland					Other countries						
	No. of patients: 1970–2010^a^	Prevalence per 100,000 live births^b^	Prevalence (number per live birth)	Poisson 95 % confidence interval	Carrier frequency^c^ ×10^3^	The Netherlands	Germany	Australia	Norway	Denmark	Sweden	Czech Republic
						Prevalence per 100,000 live births						
MPS I	48	0.22	2.2 × 10^−6^	1.4–3.0	2.96	1.19	0.69	–	1.85	0.54	0.67	0.72
MPS II	99	0.46	4.6 × 10^−6d^	3.8–5.4	4.28	0.67	0.64	0.31	0.13	0.27	0.27	0.43
MPS III (all types)	186	0.86	8.6 × 10^−6^	7.8–9.4	5.84	1.89	1.57	1.71	0.27	0.43	0.67	0.91
MPS IV (A + B)	31	0.14	1.5 × 10^−6^	0.6–2.2	2.36	0.36	0.38	0.15 (A)	0.76	0.48	0.07	0.73
MPS VI	5	0.0132	0.23 × 10^−6^	0.06–1.0	0.73	0.15	0.23	0.31	0.07	0.05	0.07	0.05
MPS VII	0	0	–	–	–	0.24	0	–	–	–	–	0.02
MPS IX	0	0	–	–	–	–	0	–	–	–	–	–
MPS unspecified^e^	23	0.11	1.1 × 10^−6^	0.2–1.9	2.09	–	–	–	–	–	–	–
MPS all types	392	1.81	18.1 × 1^−6^	17.3–18.9	8.45	4.5	3.53	3.34	3.08	1.77	1.75	3.72

^a^Total number of patients diagnosed between 1970 and 2010; in the case of MPS VI, between 1983 and 2010

^b^Data for prevalence calculations are shown in Table 2

^c^Carrier per live birth

^d^Male live births only

^e^Unspecified cases of mucopolysaccharidosis were diagnosed on the basis of analysis of glycosaminoglycans excreted in urine combined with the evaluation of clinical data. No material was available for enzyme and DNA analysis

Estimated live births from 1970 to 2010 in Poland: 21,686,890

To date, no individuals in the Polish population have been diagnosed with MPS VII or MPS IX.

The incidence and prevalence rates for MPS VI in comparison with other countries are presented in Table 2.

**Table 2:** Prevalence and incidence rates of MPS VI

Country	Incidence per 100,000 live births
Poland	0.0363
Belarus	0.3776
Estonia	0.4005
Lithuania	0.6440
The Netherlands	0.15
Germany	0.23
Australia	0.43
Norway	0.07
Denmark	0.05
Sweden	0.07
Czech Republic	0.05
Portugal	0.42

## Discussion

The overall birth prevalence of mucopolysaccharidoses in the Polish population (1.81 per 100,000 live births), which represents a Central European population, is lower when compared to the prevalences reported for other European countries, such as the Netherlands (4.5 per 100,000 live births), Czech Republic (3.72), and Germany (3.53). However, it is similar to countries such as Denmark (1.77) and Sweden (1.75) (Poupetová et al. 2010; Poorthuis et al. 1999; Malm et al. 2008; Baehner et al. 2005). The fact that there is only one center involved in the enzymatic diagnosis of mucopolysaccharidoses in Poland greatly facilitated the collection of data for this study and, as a consequence, we have a high level of confidence that few diagnoses were missed. Obviously, it is possible that there are some individuals at the less severe end of the clinical spectrum with some disorders, particularly in the adult population, in whom mucopolysaccharidosis was not diagnosed. Also, the unavailability of some diagnostic methods such as enzyme assay during the study period (some methods were introduced as late as the 1990s) may explain the lower prevalence rates.

**Fig. 2:**
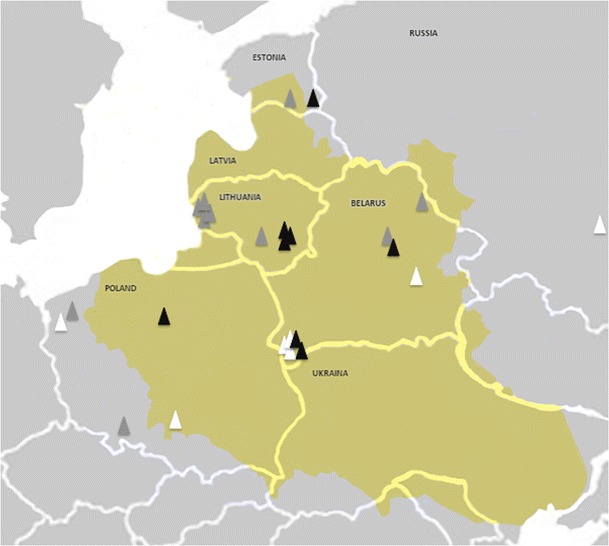
The borders of Poland and neighboring countries: the Polish–Lithuanian Commonwealth (years 1569–1686) and after World War II with geographical distribution of MPS VI patients in the present series. The *black triangles* refer to patients homozygous for p.R152W mutation; the *gray triangles* refer to families with patients heterozygous for p.R152W mutation; the *white triangles* refer to families with patients with other mutations

Different frequencies of the various forms of mucopolysaccharidosis were observed. In the Polish population, MPS III (all types) was the most frequently diagnosed (0.86 per 100,000 live births), followed by MPS II (0.46) and MPS I (0.22). In almost all published populations, Sanfilippo syndrome (MPS III) represents the most prevalent mucopolysaccharidosis. The highest prevalence was found by van de Kamp in the Netherlands (van de Kamp 1979), but it was not confirmed by a later study by Poorthuis et al. (1999), which found a crude rate of 1.89 MPS II cases in 100,000 live births. An unusually high prevalence rate of MPS II has been detected in the Ashkenazi Jewish population (Diamond 1994), while the highest prevalence of MPS I was found by Nelson in Northern Ireland (1.6 in 100,000 live births (Nelson 1997). Mucopolysaccharidosis types VI and VII belong to the less frequent mucopolysaccharidoses in most populations except for Brazil (Coelho et al. 1997). The MPS VI incidence values for Poland were the lowest of all the studies previously published so far. Interestingly, in our previous study, we found high incidence rates for MPS VI in countries neighboring with Poland (Jurecka et al. 2014). In fact, they were the highest in Europe among those reported so far. The results for Poland showed the lowest incidence rate published, except for the data of Nelson et al. (2003). The observed high p.R152W carrier frequency (0.6 %) in the Lithuanian population indicates a possible founder effect in this region (Jurecka et al. 2012). The high prevalence of this mutation (42–43 %) observed in the whole series, as well as in the Slavic origin of the majority of patients homozygous for this mutation, may suggest that p.R152W is of Slavic and not Lithuanian origin. Poland, Belarus, and Baltic States (Lithuania, Latvia, and Estonia) are located next to each other and form a belt south-east of the Baltic Sea. Historically, in the 17th century, this area consisted of one administrative unit. The Polish–Lithuanian Commonwealth was a dualistic state of Poland and Lithuania ruled by a common monarch. It was the largest and one of the most populous countries of 16th- and 17th-century Europe, with some 400,000 square miles (1,000,000 km) of territory (Fig. 2). At the end of World War II, Poland’s territory decreased from about 380,000 km^2^ before the war to about 312,000 km^2^ at present, and its borders were shifted markedly. As a result, Poland lost 50 % of its pre-war territory in the east and received about 35 % of its territory in the west. The territory in the west, which was returned to Poland after the war, was settled mainly by Poles expelled from the lost eastern territories, as well as from other regions of the country (Encyclopaedia Britannica Online 2009). Because of this, the population in the western part of Poland is very heterogenous (Tylki-Szymańska et al. 2001). This is in contrast to the population in Lithuania and Belarus. As our analysis showed, 25 % of the MPS VI patients in Lithuania are of Polish origin/ethnicity. This is likely due to the fact that a number of Poles were unable to relocate to the newly formed Poland right after the war and, thus, remained in what is present-day Lithuania. Moreover, the reported percentage of patients with Polish origin may be underrepresented, as many Polish families left behind in eastern land were pressured to hide their ethnicity. Interestingly, two-fifths of MPS VI patients identified in Poland have their family roots from the area of Vilnius, Lithuania. Unfortunately, the data from Ukraine were not available for our study. The precise estimation of incidence in case of orphan diseases like MPS VI can be difficult, as patients with attenuated phenotype may stay symptom-free and undiagnosed for a long time. It is worth mentioning that the p.R152W mutation has been associated with an attenuated MPS VI phenotype, which is often difficult to recognize and may be easily overlooked (Jurecka et al. 2011, 2012). This may additionally lead to an underestimation of the frequency of this type in this region. Interestingly, in 2013, two more MPS VI patients were diagnosed in Poland, one homozygous and one heterozygous for the p.R152W mutation. Similarly to the previous cases, the families of both patients originate from Vilnius/northern Ukraine.

In summary, individually, mucopolysaccharidoses are rare genetic diseases. However, as a group, they represent an important health problem in Poland. The prevalence data should be of interest to clinical geneticists, health care authorities, patients and their families, patient societies, and laboratories involved in the diagnosis of mucopolysaccharidoses. In addition, it provides useful information for decision-makers when it comes to estimating the social and genetic burden of these diseases to society or to considering the implementation of newborn screening programs for mucopolysaccharidoses. Prevalence rates studies are a prerequisite to health economic calculations arising from the introduction of expensive therapies like ERT.
